# Meta‐epidemiological study on the publication rate of South American countries' systematic reviews of interventions registered in PROSPERO

**DOI:** 10.1002/cesm.70002

**Published:** 2024-09-11

**Authors:** Mariana A. Burgos, Diego Ivaldi, Gisela Oltra, Camila M. Escobar Liquitay, Luis Garegnani

**Affiliations:** ^1^ Department of Research Instituto Universitario Hospital Italiano de Buenos Aires Almagro Argentina

**Keywords:** intervention studies, meta‐epidemiology, PROSPERO registry, publication bias, South America, Systematic reviews

## Abstract

**Objectives:**

To assess the publication rate and time from registration to publication of systematic intervention reviews registered in PROSPERO originated from South American countries in 2020.

**Study Design and Setting:**

Cross‐sectional study. We searched PROSPERO for protocols of systematic reviews of interventions with affiliation in South America during 2020. We randomly extracted 10% and searched databases to identify their publication status.

**Results:**

We identified 1361 intervention systematic reviews with South American affiliation registered in PROSPERO during 2020. We assessed a random sample of 10% (*n* = 135). The publishing rate in indexed journals was 36.9% (*n* = 41). The median time to publication was 1.6 years (IQR 0.9–2.1).

**Conclusion:**

The publication rate of South American PROSPERO registers is low. These findings emphasize the need for further efforts to improve publication rates and increase the visibility of South American research in the global scientific community.

## 1 BACKGROUND

Systematic reviews (SRs) form the basis of evidence‐based practice, allowing critical analysis of scientific research and its applicability in health care, improving clinical recommendations' quality and patient care [1]. The number of published SRs is constantly increasing; however, their credibility depends on the quality of the report [2]. Among the instruments that evaluate the quality of the reports, the AMSTAR‐2 (A Measurement Tool to Assess Systematic Reviews‐2) tool proposes registering protocols before starting the review [3]. Registering SR protocols promotes transparency, favors the quality of reviews, reduces redundancy, and optimizes resource allocation [4, 5], thus improving the reliability and validity of the findings, ultimately strengthening the evidence base for informed decision‐making.

Despite these advancements, a substantial disparity in scientific knowledge production persists, characterized by a low rate of publications in indexed journals and limited research citations from low‐ and middle‐income countries (LMICs) [6, 7, 8]. This disparity leads to the underrepresentation of developing countries in biomedical publications, creating an inequity in health research [9, 10] and constituting a challenge that less wealthy countries face in conducting and disseminating SRs effectively [11, 12].

Some factors identified in the literature contributing to the lack of publication in LMICs are lack of research funding [13, 14], language barriers [15, 16], limited access to databases and published articles in renowned scientific journals (a critical issue in the case of SRs) and the absence of institutional and government policies that encourage research. Furthermore, all these issues may delay knowledge production and the publication and communication of local results. Minh et al. identified that the most substantial factors associated with the publication of a systematic review protocol were the corresponding authors' registry in developed countries and the country being English‐speaking [17]. Runjic et al. [18] reported that 55% of the SRs registered in PROSPERO came from developed countries and that 54% of the reviews finally published came from these same countries.

The potential underrepresentation of the publication of South American SRs may impact the dissemination of research results. This disparity in the publication of scientific evidence at the regional level hampers evidence‐based decision‐making, potentially compromising the quality of healthcare and policy formulation in the region because locally conducted research correlates with a higher degree of compliance by healthcare professionals in developing countries [19]. Furthermore, it diminishes the global scientific contributions of South American researchers and professionals, thus impeding research equity.

Conducting a meta‐epidemiological study on the publication rate of SRs registered in PROSPERO is crucial to evaluating disparities in disseminating research, addressing possible underrepresentations of South American countries, and overcoming the lack of local evidence.

## 2 OBJECTIVE

To assess the publication rate and time from registration to publication of systematic intervention reviews registered in PROSPERO from South American countries. We hypothesize that the publication rate of systematic intervention reviews registered in PROSPERO from South American countries was low.

### 2.1 Methods

#### 2.1.1 Study design

We conducted a cross‐sectional study following the Strengthening the Reporting of Observational Studies in Epidemiology (STROBE) checklist [20] and the guidelines for reporting meta‐epidemiological methodology research [21].

### 2.2 Eligibility criteria

We included those PROSPERO registers for SRs meeting the following inclusion criteria:

− Assessed the effect of any intervention.
− Any health condition.
− That they had Organizational affiliation.
− The Organizational affiliation corresponds to a South American country (Argentina, Bolivia, Brazil, Chile, Colombia, Ecuador, French Guiana, Guyana, Paraguay, Perú, Suriname, Uruguay and Venezuela). We determined eligibility based on the “Organizational affiliation” and “Country” information provided in the PROSPERO registry. When this information was unavailable, we conducted a hand search to identify the lead author's institutional affiliation. To ensure accuracy, we assessed the first or corresponding author's affiliation. If there were multiple authors, we prioritized the affiliation of the lead or corresponding author as indicated in the publication.

### 2.3 Search strategy

#### 2.3.1 Identification of records in PROSPERO

We searched PROSPERO records of SRs between January 1, 2020, and December 31, 2020. We considered organizational affiliation terms for countries in the South American region: Argentina, Bolivia, Brazil, Chile, Colombia, Ecuador, French Guiana, Guyana, Paraguay, Peru, Suriname, Uruguay, and Venezuela.

We apply the following PROSPERO filters:

- Health Area of Review: Any health area.
- Type and method of the review: Intervention.
- Source of the Review: Exclude Cochrane Protocols.
- Status of the Review: Any revision status.
- Restrict search to specific fields: Intervention [IV].
- Date added to PROSPERO: Start Date: January 1, 2020 End Date: December 31, 2020. We selected this time frame because the year 2019 would not be representative of the state of publications generated in South America, as most efforts were focused on COVID‐19 research. By selecting 2020, our goal was to ensure a clearer and more stable context for our analysis.

We transferred all recovered records to an Excel spreadsheet. From these, considering the project's feasibility, we randomly selected a representative sample of 10%. We reviewed the records, considering the information requested by the PROSPERO platform, and reviewed the inclusion and exclusion criteria of the obtained sample. We performed a calibration among the authors of the team with 5% of the records, evaluating homogeneity in the eligibility process.

#### 2.3.2 Data extraction from PROSPERO SRs records

We extracted the following data from the PROSPERO records: PROSPERO identification number, organizational affiliation, country, number of authors, population included, health topic, language of publication, and date of registration. We performed a calibration among the authors of the team with 5% of the records, evaluating homogeneity in the data extraction process.

#### 2.3.3 Identification of complete SRs published in indexed journals

We manually tracked the completion and publication of included registers in an indexed journal using at least one of the following criteria:

- Search by each record's unique PROSPERO identification number (CRD: Centre for Reviews and Dissemination).
- Search by title of the protocol registered in PROSPERO.

We searched the following databases: MEDLINE (Pubmed), Lilacs, Embase, Scopus, (Elsevier) Isi Web Of Science (WOS), and Google Scholar.

We defined the status of a record as **“published”** if the unique PROSPERO CRD record number matched that of the publication. If the CRD number was not available in the publication, we considered the registration and publication in PROSPERO to be the same if at least one of the authors, the title, and the objective were the same and the time window (post‐2020) matched. Furthermore, we defined a review as “published” if it had been published in an indexed journal. We defined a journal as **“indexed”** if it corresponded to a periodical publication that had been evaluated and selected for inclusion in a specific database or index and was retrievable in at least one of the following reference databases: MEDLINE, LILACS; Embase, Scopus, or WOS [22, 23].

We defined as **“unpublished”** those registers not retrieved by any of the proposed strategies and those we found published in institutional repositories or nonindexed journals.

#### 2.3.4 Data extraction from SRs published in indexed journals

We extracted the following data from publications in indexed journals: organizational affiliation, health topic, the included population, number of authors, language of publication, date of publication, and type of intervention. To determine whether the organizational affiliation corresponded to a university, a health‐related institution, a hospital, or another type of organization, we conducted a manual search using the ítem “Organizational affiliation information” declared by the authors in the PROSPERO registry. When this information was unavailable, we performed a manual search to identify the institutional affiliation of the lead author or corresponding author. For publications with multiple authors, we prioritized the affiliation of the primary or corresponding author as indicated in the publication. We solved all discrepancies through discussion and consensus with an information analyst and a professional expert.

We defined the Publication Time as the time from the first PROSPERO registration date until the first publication date of the completed SR (in years).

Five authors participated in the data extraction process from PROSPERO SR records, the identification of complete SRs, and the extraction of data from these SRs. All authors were thoroughly trained in these procedures and calibrated among themselves to ensure the precision and reproducibility of the search and data extraction process.

#### 2.3.5 Statistical analysis

We reported continuous variables as means and standard deviations or medians and interquartile ranges according to the distribution, analyzed by visual inspection of histograms, standardized normal probability plots, and the Shapiro‐Wilk test. We reported categorical variables as absolute numbers and proportions. We reported the proportion of studies published a percentage. We reported the time to publication in years. We used STATA 16.0 software for the statistical analysis (StataCorp LLC).

## 3 RESULTS

**Figure 1:**
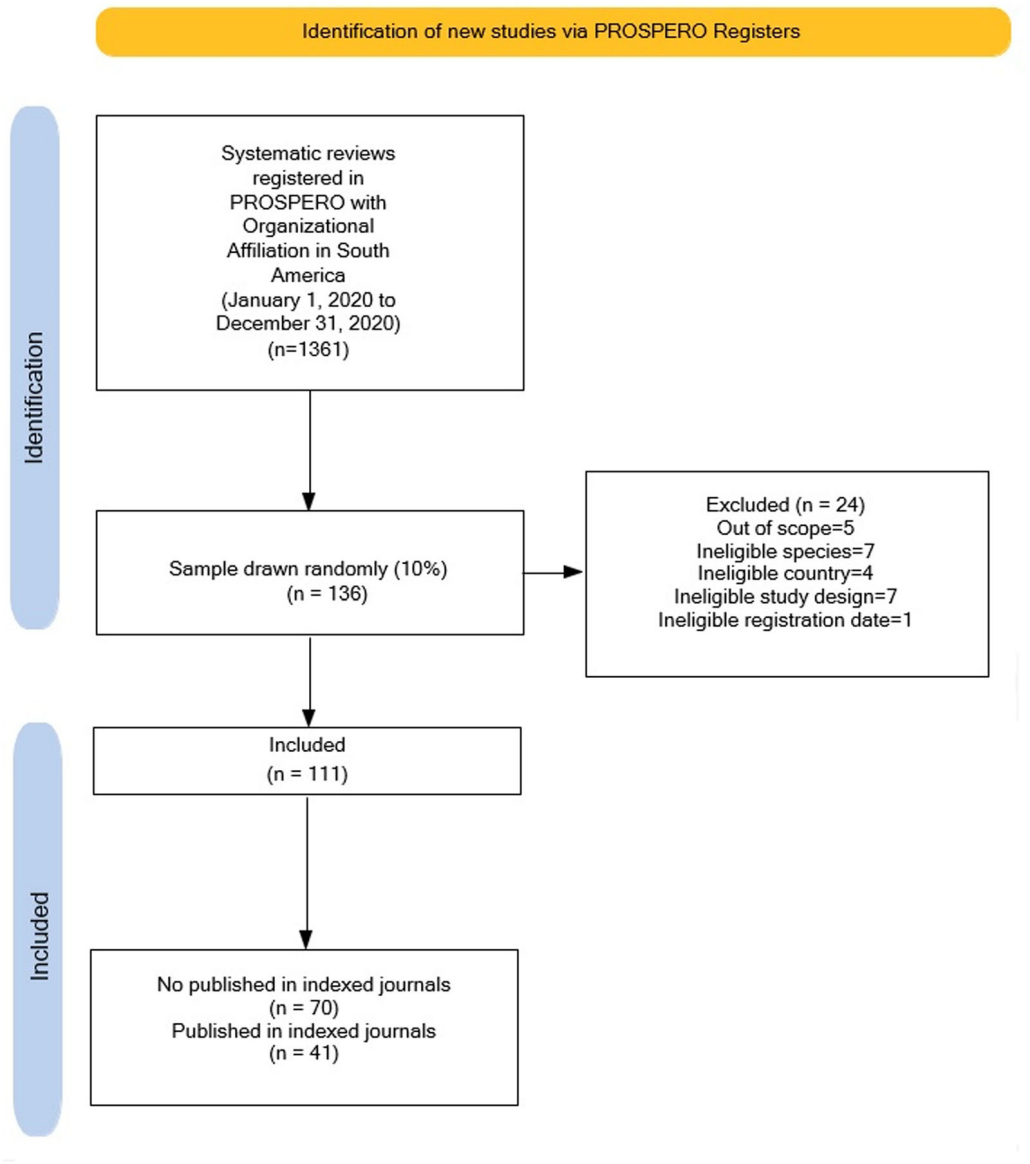
Study flow diagram.

We identified 1361 systematic intervention review protocols registered in PROSPERO through our search strategy. We randomly selected 135 studies (equivalent to 10% of the total). We excluded 24 of these for different reasons. See the flow diagram in Figure 1. See the list and characteristics of excluded studies in the Supporting Information material.

Among the 111 included SRs protocols registered in PROSPERO, countries with the most registries, ranked by frequency, were Brazil with 95 (86%), Colombia with 7 (6%), Chile with 6 (6%), Peru with 2 (2%), and Ecuador with 1 (0.1%). None of the other South American countries recorded any SR protocol in 2020. See the descriptive characteristics of these records in Table 1.

**Table 1:** Characteristics of included PROSPERO registers.

Characteristics	Summary statistics
Organizational affiliation	
University	87/111 (78.3%)
Other organization	15/111 (13.5%)
Institutions related to health	5/111 (4.5%)
Hospitals	4/111 (3.6%)
Topic	
Physical rehabilitation	33/111 (29.7%)
Oral health	30/111 (27%)
Chronic diseases	10/111 (9%)
Infectious diseases	7/111 (6.3%)
Oncology	7/111 (6.3%)
Mental health	6/111 (5.4%)
Pediatric care	3/111 (2.7%)
Women's health	2/111 (1.8%)
Other	13/111 (11.7%)
Number of authors	
1	5/111 (4.5%)
2–3	28/111 (25.2%)
4–5	54/111 (48.7%)
6–7	20/111 (18%)
≥8	4/111 (3.6%)
Main language	
English	108/111 (97.3%)
Portuguese (Brazil)	1/111 (0.9%)
Spanish	1/111 (0.9%)
Not reported	1/111 (0.9%)
Population	
Children and adolescents (≤18 years)	8/111 (7.2%)
Adults (>18 years)	40/111 (36%)
Mixed (no age limit)	25/111 (22.5%)
Not reported or not applicable	38/111 (34.2%)
Type of intervention	
Nonpharmacological	79/111 (71.2%)
Pharmacological	30/111 (27%)
Both	2/111 (1.8%)

Of the 111 PROSPERO records included, 70 (63%) were not published, among which 62 (55%) were not found with our search methodology and 8 (7%) were located as publications in institutional repositories or nonindexed journals.

Finally, we analyzed 41 articles (36.9%) that were published in indexed journals. The median time to publication was 1.61 years (IIQ 0.9–2.1). Among them, Brazil, Colombia, Chile and Peru were the countries with the most published records, with 33 (80%), 4 (9.7%), 3 (7.3%) and 1 (2.4%), respectively. The main language of publication was English, representing 100% of the studies (41/41). No studies were reported in Portuguese (Brazil) or Spanish.

See the characteristics of the SRs published in indexed journals in Table 2.

**Table 2:** Characteristics of the PROSPERO registries of SRs with publication in indexed journals.

Characteristics	Summary statistics
Organizational affiliation	
University	37/41 (90%)
Hospitals	3/41 (7.3%)
Institutions related to health	1/41 (2.5%)
Topic	
Oral health	17/41 (41.4%)
Chronic diseases	6/41 (14.6%)
Physical rehabilitation	5/41 (12.1%)
Oncology	4/41 (9%)
Mental health	4/41 (9%)
Pediatric care	2/41 (5%)
Other	3/41 (7.3%)
Number of authors	
1	1/41 (2.5%)
2–3	9/41 (22%)
4–5	19/41 (46%)
6–7	10/41 (24%)
≥8	1/41 (2.5%)
Population	
Children and adolescents (≤18 years)	3/41 (7.3%)
Adults (>18 years)	13/41 (32%)
Mixed (no age limit)	4/41 (9.8%)
Not reported or not apply	21/41 (51%)
Type of intervention	
Nonpharmacological	28/41 (68%)
Pharmacological	11/41 (29%)
Both	5/41 (12%)

## 4 DISCUSSION

We found that only a third of SRs registered in PROSPERO during 2020 were published. A study evaluating the publication status of the PROSPERO registries related to anesthesia and pain as a topic reported a publication rate of 53% [18], with Brazil as the only South American country, which ranked sixth with a total of 68 SRs (7.4%) published between 2017 and 2018. While the focus of this study differs from ours, the consistently low publication rate for South American countries aligns with our findings. Since 2004, Sumathipala [6] assessed publication biases by analyzing the contribution of developing countries in medical literature published in high‐impact journals and found that only 6.5% of the articles published in these journals came from these countries. Although the focus of this study differs from ours, the rate of consistently low publication for South America is in line with our findings, highlighting the underrepresentation of developing countries in global scientific literature, reducing the visibility of South America internationally. The low rate of South American research publications may result in a limited understanding of the region's specific health problems, which has significant implications for public health, decision‐making, and local scientific advancement.

We found a longer time to publication than that reported by Borah et al. in which a median of 1.4 years was estimated until the publication of an SR registered in PROSPERO [24]. This finding is consistent with another study conducted in 2018, focusing on anesthesia and pain protocols registered in PROSPERO, which estimated the time to publication to be 1.3 years [18]. However, according to a study by Tricco et al., reviews conducted by the Cochrane Collaboration in 2000–2001 (excluded from this review) had a median publication time of 2.4 years [25]. This could be related to the rigor of the Cochrane Collaboration's numerous review processes. Our study showed that the time to publication was longer than other non‐Cochrane reviews. The delay in publishing SRs in low‐resource or developing countries hinders timely access to critical medical insights, impacting healthcare decision‐making, research progress, and the ability to address evolving health challenges efficiently.

The preeminence of Brazil in both registration and publication underlines the need to generate local initiatives that promote research in other South American countries. In 2017, Page et al. [4] carried out a methodological review on a random sample of 150 SRs registered in PROSPERO, in which Brazil ranked fourth (11%) in terms of registration frequency, sharing the position with China. There was very low representation (less than 5 reviews per country for the remaining countries).

We found that the most frequently reported organizational affiliation in PROSPERO registration (and also in the subsequent publication) was the university, suggesting that proposals for SRs might be primarily led by academic institutions in our region. This could be attributed to the fact that, in South America, universities predominantly offer access to libraries, academic databases, and essential resources for conducting SRs. Furthermore, these institutions provide funding opportunities and research infrastructure, making them a favorable environment for undertaking such studies.

We found that both registration and publication mostly involved adult participants, which could lead to an underrepresentation of pediatric and adolescent populations. This is consistent with other studies, showing that the incorporation of vulnerable populations into intervention studies is scarce [18, 26].

About language, all of the RS publications in our study were published in English. The dominance of English as the publication language raises questions about inclusivity and accessibility in the dissemination of SRs and potentially indicates a limited representation of researchers who do not communicate in English. This is consistent with the literature in which English dominates the publications at 87% and no other individual language reaches 2% [27].

The prevalent theme in the SRs we analyzed was related to Physical Health and Rehabilitation. While this issue is globally relevant, it is not uniquely pertinent to South America. By focusing our search solely on reviews of intervention studies, we may have inadvertently excluded many topics specific to South America, such as infectious diseases prevalent on the continent. This lack of representativeness regarding topics of regional interest highlights the need for further research to provide a deeper understanding of the unique health challenges South American populations face. Addressing these specific health problems would not only enrich the existing literature but also aid in the better allocation of resources. We plan to conduct these additional analyses in future studies to address these gaps.

Our study is not free from limitations. First, we took the 2 years after 2020 to evaluate the publication of the studies registered in PROSPERO and this time window could be shorter than that required for SRs to be completed and published, as shown in the article by Tricco et al. [25]. Second, 2020 coincided with the COVID‐19 pandemic, and this global health crisis also had profound effects on health systems worldwide. Consequently, the data from 2020 may not accurately reflect the landscape of SRs generated in South America, however, in selecting 2020, our goal was to ensure a slightly clearer and more stable context for our analysis [28]. Third, we carried out this study considering SRs of intervention, this limited our field of study and the decision made by feasibility could condition the generalization of our findings. As a final consideration, we understand that it would have been of great interest to explore characteristics such as the impact factor of the journals in which the publication was published, but unfortunately, this was not possible due to the high variability in the way in which the journals report it. Some journals used the Clarivate index, others used Scopus or the H‐index, some did not have it updated, and in some cases, we could not find any impact factor. This heterogeneity made their categorization and comparison difficult. “Inconsistency and difficulty in finding information on impact factors can hinder the visibility, recognition, and advancement of research from developing countries, perpetuating existing disparities in the global academic and scientific community.

## 5 IMPLICATIONS FOR PRACTICE AND RESEARCH

Our study objectively quantifies the low global visibility of South American research. SRs serve as a critical resource for the development of clinical practice guidelines, facilitating diverse healthcare stakeholders to make informed decisions in their daily clinical practices. The underrepresentation of South American countries in indexed publications exemplifies the challenges researchers from developing countries face in disseminating local research results. This disparity has a direct impact on communities that benefit greatly from such crucial information [14]. Our findings highlight the urgent need to promote initiatives aimed at improving the visibility of local research within the global scientific community through collaborative efforts.

## 6 CONCLUSIONS

The publication rate of SRs intervention registered in PROSPERO originated from South American countries is low. These findings emphasize the need for further efforts to improve publication rates and increase the visibility of South American research in the global scientific community.

## AUTHOR CONTRIBUTIONS

**Mariana A. Burgos**: Conceptualization; data curation; formal analysis; methodology; validation; writing—original draft. **Diego Ivaldi**: Data curation; formal analysis; validation. **Gisela Oltra**: Data curation; formal analysis; validation. **Camila M. Escobar Liquitay**: Formal analysis; methodology; validation. **Luis Garegnani**: Methodology; project administration; validation; visualization; writing—review and editing.

## CONFLICT OF INTEREST STATEMENT

The authors declare no conflict of interest.

## PEER REVIEW

The peer review history for this article is available at https://www.webofscience.com/api/gateway/wos/peer-review/10.1002/cesm.70002.

## Supporting information

Supporting information.

## Data Availability

The data that support the findings of this study are available from the corresponding author upon reasonable request.
