# Supplementary material for: Meta‐epidemiological study on the publication rate of South American countries' systematic reviews of interventions registered in PROSPERO
Source: Cochrane Evid Synth Methods. 2024 Sep 11;2(9):e70002. doi: 10.1002/cesm.70002 (PMC11795941; doi:10.1002/cesm.70002)
Supplement: Supplementary file 1 — Supporting information. [file CESM-2-e70002-s001.docx]

**Supplementary Material**

**Characteristics of excluded studies**

| **Reference** | **Title** | **DOI** | **Detailed Reason for exclusion** |
| --- | --- | --- | --- |
| CRD42020152181 | Comparative effectiveness and cost-effectiveness of ACTH versus prednisolone for the treatment of infantile spasms. A meta-analysis and cost-effectiveness study. | <https://www.crd.york.ac.uk/prospero/display_record.php?RecordID=152181> | The country of origin was not South American |
| CRD42020205555 | A systematic review and meta-analysis on the clinical effectiveness of topical hyaluronic acid in oral ulcers. | <https://www.crd.york.ac.uk/prospero/display_record.php?RecordID=205555> | The country of origin was not South American |
| CRD42020209352 | Effects of plant / herbal extracts in the treatment of recurrent aphthous stomatitis: a systematic review | <https://www.crd.york.ac.uk/prospero/display_record.php?RecordID=209352> | Study carried out in animal models |
| CRD42020205516 | A systematic review and network meta-analysis of the predictive validity of the animal models of depression | <https://www.crd.york.ac.uk/prospero/display_record.php?RecordID=205516> | Study carried out in animal models |
| CRD42020208167 | The effect of rapamycin and its analogues on age-related musculoskeletal conditions | <https://www.crd.york.ac.uk/prospero/display_record.php?RecordID=208167> | The country of origin was not South American |
| CRD42020204845 | Peptides from animal origin: A systematic review and in silico integrated approach on chemical structures, enzymatic interactions and effects on skin wounds | <https://www.crd.york.ac.uk/prospero/display_record.php?RecordID=204845> | Study carried out in animal models |
| CRD42020202446 | Experiences of stigma of people with overweight and obesity in Latin America, Africa and Asia: a systematic review. | <https://www.crd.york.ac.uk/prospero/display_record.php?RecordID=202446> | Systematic non-intervention review |
| CRD42020208658 | Effect of diabetes mellitus on epididymal morphology: a systematic review. | <https://www.crd.york.ac.uk/prospero/display_record.php?RecordID=208658> | Study carried out in animal models |
| CRD42020205390 | Systematic review and meta-analysis of mitochondrial-related gene expression in the heart after doxorubicin treatment: a comparative study | <https://www.crd.york.ac.uk/prospero/display_record.php?RecordID=205390> | Study carried out in animal models |
| CRD42020204776 | Assesment of anti-nociceptive properties of ômega-3 supplementation in animal models of inflammatory and neuropathic pain: a systematic review | <https://www.crd.york.ac.uk/prospero/display_record.php?RecordID=204776> | Study carried out in animal models |
| CRD42020203558 | Vitamin D and Mental Health Outcomes: A Protocol for an Umbrella Systematic Review | <https://www.crd.york.ac.uk/prospero/display_record.php?RecordID=203558> | No systematic review |
| CRD42020203359 | Influence of thyroid hormone on the nutritional recovery of children with different states of malnutrition. | <https://www.crd.york.ac.uk/prospero/display_record.php?RecordID=203359> | Systematic non-intervention review |
| CRD42020205057 | Electromyographic evaluation of masticatory muscle in children and adolescents with posterior crossbite: a systematic review. | <https://www.crd.york.ac.uk/prospero/display_record.php?RecordID=205057> | No systematic review |
| CRD42020207740 | A living systematic review of clinical trial protocols for novel COVID-19 vaccines: the participation of pregnant women and low- and middle-income countries | <https://www.crd.york.ac.uk/prospero/display_record.php?RecordID=207740> | Systematic non-intervention review |
| CRD42020152041 | Sugar intake and risk of colorectal cancer: a systematic review and meta-analysis | <https://www.crd.york.ac.uk/prospero/display_record.php?RecordID=152041> | No systematic review |
| CRD42020201502 | Transmigrated lower canines: Do the challenging treatment with orthodontic traction protocols give an appropriate occlusion and stability? A systematic review. | <https://www.crd.york.ac.uk/prospero/display_record.php?ID=CRD42020201502> | No systematic review |
| CRD42020205209 | Hysteroscopic metroplasty of septate uterus and reproductive outcomes - a systematic review with meta-analysis. | <https://www.crd.york.ac.uk/prospero/display_record.php?RecordID=205209> | The country of origin was not South American |
| CRD42020205563 | What is the best technique for fixing the mesh on the laparoscopic surgery for inguinal hernia? A systematic review of systematic reviews | <https://www.crd.york.ac.uk/prospero/display_record.php?RecordID=205563> | Systematic non-intervention review |
| CRD42020206390 | Heterogeneity in the effect size of vaccines to prevent COVID-19 in randomized clinical trials from less developed and more developed countries. A meta-epidemiological study | <https://www.crd.york.ac.uk/prospero/display_record.php?RecordID=206390> | Systematic non-intervention review |
| CRD42020207866 | Cervical and scapular myofascial pain and trigger point muscle injections: Systematic review - network meta-analysis | <https://www.crd.york.ac.uk/prospero/display_record.php?RecordID=207866> | No systematic review |
| CRD42020205316 | Substance use among the elderly during the COVID-19 pandemic: a review of the literature and an overview | <https://www.crd.york.ac.uk/prospero/display_record.php?RecordID=205316> | No systematic review |
| CRD42020209184 | Heterogeneity in the effect sizes of convalescent plasma transfusion for treating patients with COVID-19 in randomized clinical trials from less developed and more developed countries: a meta-epidemiological assessment | <https://www.crd.york.ac.uk/prospero/display_record.php?RecordID=209184> | No systematic review |
| CRD42020206140 | Effectiveness of Pilates to improve balance and posture in the elderly: a meta-analytical study of potential training moderators | <https://www.crd.york.ac.uk/prospero/#searchadvanced> | Study carried out in animal models |
| CRD42020152011 | Disinfection the pacifiers: How to make it? | <https://www.crd.york.ac.uk/prospero/display_record.php?RecordID=152011> | Not registered in 2020 |
